# The association of the triglyceride-glucose index and its changes with 5-year all-cause mortality in patients with depression

**DOI:** 10.3389/fpsyt.2025.1672186

**Published:** 2025-10-08

**Authors:** Xiao-Jiao Cui, Bo Xie, Yu-Fei Zhou, Xiao-Qing Yi

**Affiliations:** ^1^ Department of Pharmacy, Personalized Drug Therapy Key Laboratory of Sichuan Province, Sichuan Academy of Medical Sciences & Sichuan Provincial People’s Hospital, School of Medicine, University of Electronic Science and Technology of China, Chengdu, China; ^2^ Department of Cardiology, Chengdu Integrated TCM & Western Medicine Hospital, Chengdu, China

**Keywords:** triglyceride-glucose index, changes, all-cause mortality, depression, risk factor

## Abstract

**Background:**

Depression, as the primary contributor to the global disease burden caused by mental disorders, necessitates the urgent discovery of new biomarkers to predict patient prognosis, thereby facilitating early treatment. The triglyceride glucose index (TyG), which has a close relationship with insulin resistance (IR), systemic inflammation, and other factors, could potentially serve as a valuable biomarker for determining depression severity. However, the association of the TyG index and its changes with the long-term prognosis of depression remains unexplored. This article aims to evaluate whether the levels and changes in the TyG index can predict long-term mortality in patients suffering from depression.

**Methods:**

A retrospective cohort study was conducted based on the MIMIC-IV (Medical Information Mart in Intensive Care IV) database, involving 1388 patients. Among them, 1120 patients had only one TyG index value during hospitalization, while 266 patients had two or more TyG index values, all of which were included in the analysis. The primary endpoint was 5-year all-cause mortality rate. Restricted cubic spline analysis was also used to evaluate any potential nonlinear correlations. Propensity score matching was performed to reduce any potential baseline bias. Cox proportional hazards analyses were used to adjust for confounders. The Kaplan-Meier method was utilized to compute the cumulative curve.

**Results:**

The entire cohort exhibited a 5-year all-cause mortality rate of 25.8% (358/1388), with rates of 23.8% (267/1120) and 34% (91/268) in the TyG and TyGVR groups, respectively. According to a multivariate Cox regression model, an increase in TyG elevated the 5-year all-cause mortality risk among depression patients (HR, 1.31; 95% CI, 1.1-1.56; P=0.002). When TyG was treated as a categorical variable, Quartile 4 demonstrated a 61% higher risk of 5-year all-cause mortality in depression patients compared to Quartile 1 (HR, 1.61; 95% CI, 1.15-2.25; P=0.005). Restricted cubic spline analysis revealed a linear relationship between TyG and 5-year all-cause mortality across both the entire and matched cohorts (P values for non-linearity were > 0.05). The Kaplan-Meier curves indicated that patients with elevated TyG experienced significantly higher 5-year all-cause mortality rates in both the entire and matched cohorts (P=0.00041, P=0.035, respectively). A linear association (non-linear P=0.953) was also observed between TyGVR and 5-year all-cause mortality risk. The analysis results across the subgroups were consistent, with no interactions detected (interaction P-values > 0.05).

**Conclusions:**

The TyG index is linked with a 5-year all-cause mortality risk among patients suffering from depression. The dynamic fluctuations in the TyG index could potentially offer more substantial insights in identifying patients at high risk for all-cause mortality.

## Introduction

Depressive disorders, leading contributors to the global disease burden caused by mental disorders, have emerged as significant public health issues worldwide (1). The global prevalence of depression has surged by nearly 50% over the past three decades, currently affecting over 258 million individuals across all age groups (2). Major depressive disorder (MDD) is widely recognized as one of the most pressing mental health issues (3). Individuals suffering from depression are at an increased risk of developing cardiovascular diseases, and the severity of depression correlates with the risk of subsequent death and other cardiovascular events (4–8). Furthermore, depression is frequently linked with suicide (9), and given the severe harm it can inflict, no form of depression should be trivialized (3). Regrettably, most individuals with depression remain untreated or undertreated (10), often leading to prolonged suffering and reduced life expectancy. Even post-treatment, a prior study revealed that 15% of individuals with MDD committed suicide (11). The recurrence of depression varies considerably among individuals, with some experiencing recurrent episodes throughout their lives, while over half never relapse (3). As a result, personalized treatment for depression is garnering increased attention (10). The urgent need to identify new biomarkers to predict the prognosis in depression patients is evident, aiming to guide early treatment, reduce disease relapse or suicidal behavior, and thereby mitigate the progression of the personal and societal burden of depression (12, 13).

The triglyceride-glucose index (TyG), derived from the logarithmic conversion of the product of fasting glucose and triglyceride levels, is an epidemiological biomarker of metabolic dysfunction and is strongly associated with insulin resistance (IR), metabolic syndrome, and systemic inflammation (14–16). Research indicates that elevated TyG index levels are closely tied to a higher incidence of diabetes and prediabetic conditions (17, 18). Several clinical studies have confirmed a positive correlation between the TyG index and the prevalence and prognosis of cardiovascular and cerebrovascular diseases (19, 20).

Critically, a bidirectional relationship exists between major depressive disorder (MDD) and IR-related diseases (21), and MDD comorbidities involving IR are linked to greater depression severity, chronicity, and poorer treatment outcomes (6, 8, 22). This strong pathophysiological link underscores the need for robust biomarkers to quantify metabolic dysfunction in depression. However, the vast majority of existing studies have focused exclusively on the static value of the TyG index measured at a single time point. This approach overlooks the potential significance of intra-individual fluctuations in triglyceride and glucose levels over time—as reflected by the variability of the TyG index (TyGVR)—which may represent a distinct and critical aspect of metabolic pathophysiology. Emerging evidence indicates that high TyG variability is associated with adverse outcomes (23, 24). The underlying mechanisms may involve erratic metabolic control, subclinical inflammation, or impaired adaptive capacity, all of which could exacerbate the pathological processes linking depression and metabolic dysfunction. Therefore, investigating TyG variability may offer novel insights into the metabolic instability associated with depression and its long-term risks, providing a dynamic biomarker beyond single-time-point measurements.

The TyG index may serve as a valuable indicator for gauging depression severity, and several studies have investigated its correlation with depression. A large-scale cross-sectional study (25) revealed a significant likelihood of depressive symptoms among American adults with a higher TyG index. Another study (26) discovered a non-linear relationship between the TyG index and the risk of suicide attempts, demonstrating a threshold effect in Chinese patients with major depressive disorder initially treated with first-line drugs.

Nonetheless, the relationship between the TyG index and its variations with the long-term prognosis of depression has not been previously explored. This article aimed to evaluate whether the level and changes in the TyG index can predict the long-term mortality of patients with depression. The evaluation is based on data derived from the “Medical Information Mart in Intensive Care-IV (MIMIC-IV)”.

## Methods

### Data source

This study was a retrospective cohort study based on the MIMIC-IV database (27). MIMIC-IV is a publicly available database sourced from the electronic health records and record dataset of the Beth Israel Deaconess Medical Center, covering a decade of hospitalizations from 2008 to 2019. The Institutional Review Board at the Beth Israel Deaconess Medical Center granted a waiver of informed consent and approved the sharing of the research resource. Author Cui passed the online training courses and exams (certification number: 59921922). MIMIC-IV establishes a modular organization of the constituent data allowing linking of the database to external departments and distinct modalities of data which allowed us to explore the availability of out-of-hospital mortality.

### Study population

**Figure 1:**
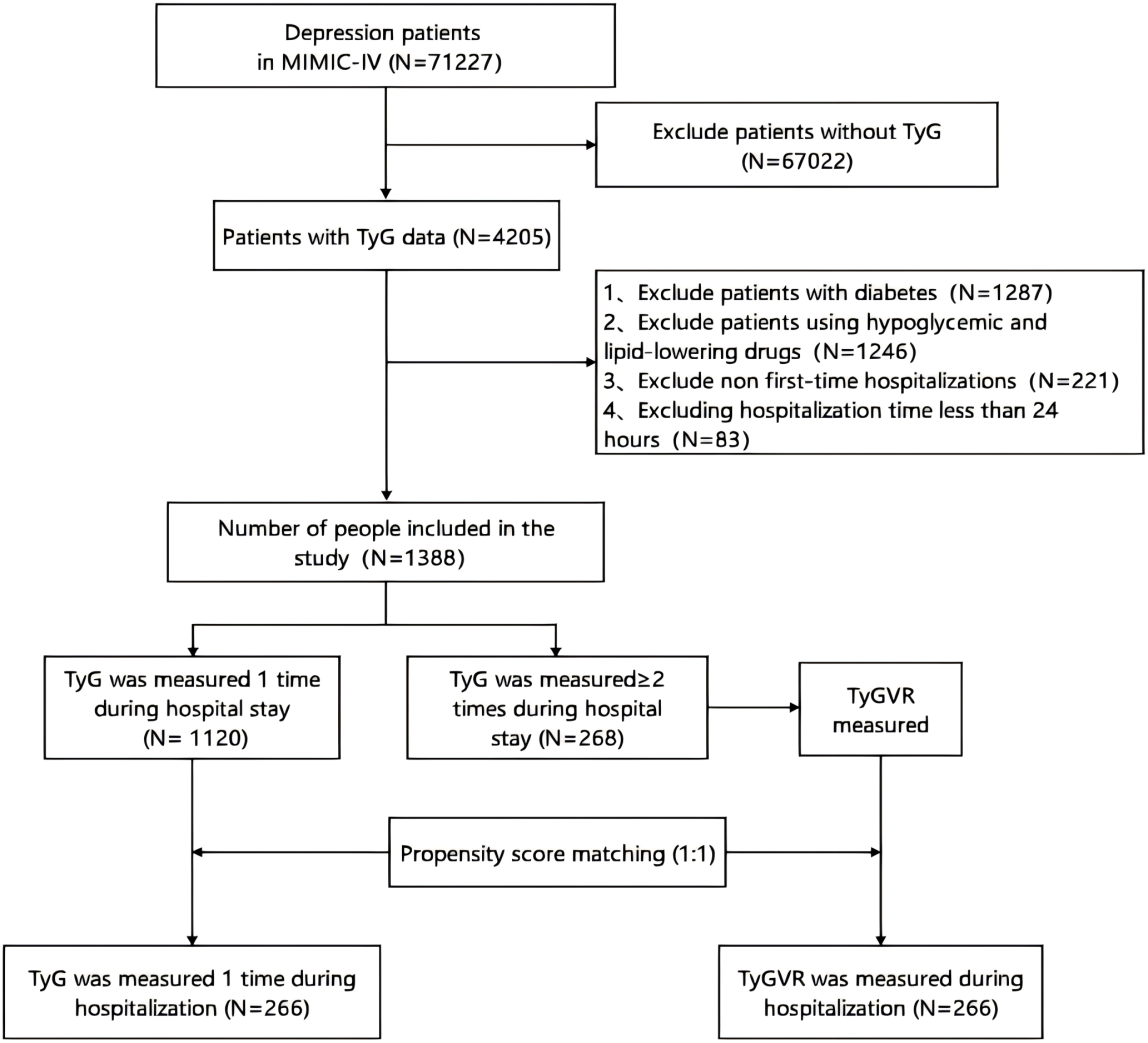
Flow chart of patient selection. Medical Information Mart for Intensive Care IV, Multiparameter Intelligent Monitoring in Intensive Care Database IV; TYG, triglyceride-glucose index; TYGVR, triglyceride-glucose index variability ratio.

Selection criteria, as well as the number of patients, were excluded at each step, were shown in **Figure 1**. Patients whose diagnostic description included “Depress”, “Severe depression with single episode”, and “Severe depression with recurrent episodes” were enrolled in the study. We excluded patients under the age of 18 years or those who had been hospitalized for less than 24 hours. If the patient has been hospitalized multiple times, only the first hospitalization will be analyzed. Among the 71,227 patients, we excluded 67,022 patients with missing TyG values, and excluded 1287 patients with pre-existing diabetes and 1246 patients using hypoglycemic or lipid-lowering agents. We then further excluded 221 patients who were not hospitalized for the first time. In addition, 83 patients were excluded because of a stay of less than 24 hours. Finally, 1388 patients were included in this cohort analysis, which was divided into two groups based on the times of measurement of TyG index during hospitalization.

### Exposure and outcomes

The exposure of this study was the TyG index and the TyG variability ratio (TyGVR) collected during hospitalization. The TyG index was calculated by the formula ln[TG (mg/dl) × FBG (mg/dl)/2] (28). TyGVR was calculated as follows (23): TyGVR=(TyG average −TyG baseline)/(TyG baseline). Fasting blood glucose and triglycerides recorded for the first time after admission were used to calculate the TyG baseline. TyG average was defined as the average value of fasting blood glucose and triglycerides recorded on multiple occasions, excluding the first time (23).

The main outcome was 5-year all-cause mortality rate. Secondary outcomes included in-hospital mortality rate and 1-year all-cause mortality rate.

### Data extraction

Baseline variables within 24 hours of admission were collected from the MIME-IV database. Patient characteristics were collected, including age, gender, race, BMI, and insurance. The following laboratory indicators were extracted: fasting plasma glucose, fasting triglycerides, total cholesterol (TC), HDL, LDL, ALT, AST, and serum creatinine. We extracted information on antidepressant use. We also extracted information about comorbidities, such as myocardial infarction, congestive heart failure, cerebrovascular disease, atrial fibrillation, hypertension, ischemic stroke, chronic lung disease, rheumatic disease, peptic ulcer disease, liver disease, kidney disease and malignant cancer. All comorbidities were identified based on ICD-9 or ICD-10 codes.

### Statistical analysis

For the baseline characteristics, mean (standard deviation [SD]) and median (interquartile range [IQR]) were used for description of normally and non-normally distributed data, respectively (29). Categorical variables were presented as counts and percentages, which compared by Pearson’s chi-squared test. Multiple imputation was used to estimate missing values for each variable in our study (30, 31). Ten patients with a BMI greater than 100 or less than 10 were considered outliers. A preliminary analysis was conducted in a matched cohort to explore the relationship between TyG index and primary and secondary outcomes. The relationship between TyGVR and main outcome was further analyzed. In order to minimize potential bias between the two study groups, we used propensity score matching to adjust variables according to the recommendations in the literature (32). The matching was constructed based on a 1:1 ratio using the nearest neighbour method with a calliper width of 0.05 without replacement. The balance of variables between the groups before and after matching was assessed using standardised mean difference (SMD), with a value of less than 0.10 indicating an appropriate balance between groups (33). Cox proportional hazards regression analyses with results expressed as hazard ratios (HR) with 95% CI were used to assess the relationship between TyG index and 5-year all-cause mortality. The statistically significant variables (P<0.05) in the univariate analysis were included as covariates in the multivariate analysis, and three multivariate models were used for TyG index and TyGVR, respectively. Restricted cubic spline was used to assess potential nonlinear relationships between the levels of TyG index and TyGVR with 5-year all-cause mortality. Additionally, subgroup analysis was conducted to explore the potential impact of variables such as gender, age, race, and BMI. These results were calculated using patient data after propensity score matching (PSM).

All the analyses were performed with the statistical software packages R(http://www.R-project.org,The R Foundation)and Free Statistics software versions 1.9.

## Results

### Cohort characteristics

This study contained 1,388 eligible subjects in total, stratified by TyG index quartile, as **Table 1** illustrates. With a mean age of 54.3 ± 18.1 years, 37.7% of the population was male and 72.3% was white. 42.1% of patients were single, and 34.1% were married. At the depressive level, diagnoses were made for 49.1% of patients with depression, 44.7% with severe depression with a single episode, and 6.3% with recurring severe depression. Compared with the Q1 group, patients in other groups are more likely to be male, with a higher BMI, and have a higher prevalence of myocardial infarction, hypertension. Patients with a higher TyG index have higher levels of glucose, cholesterol, triglycerides, low-density lipoprotein (LDH), and transaminase. In addition, longer length of stay (LOS), higher in-hospital mortality rate, 1-year all-cause mortality rate, and 5-year all-cause mortality rate were observed in the group with higher TyG index.

**Table 1:** Baseline characteristics of the entire cohort stratified by the TyG index quartiles.

Variables	Overall (n =1388)	Q1 (n=347)	Q2 (n=347)	Q3 (n=347)	Q4 (n=347)	P value
Demographics						
Age, year	54.3 ± 18.1	52.0 ± 20.3	56.1 ± 18.3	55.1 ± 17.2	54.1 ± 16.3	0.019
Gender, Male, n (%)	523 (37.7)	110 (31.7)	129 (37.2)	138 (39.8)	146 (42.1)	0.032
Race white, n (%)	1003 (72.3)	240 (69.2)	264 (76.1)	260 (74.9)	239 (68.9)	0.06
BMI, kg/m2	28.2 ± 8.2	27.4 ± 8.9	27.8 ± 8.1	28.3 ± 8.0	29.1 ± 7.4	0.043
Depression level						0.451
Depression	681 (49.1)	168 (48.4)	172 (49.6)	170 (49)	171 (49.3)	
Severe depression with single episode	620 (44.7)	148 (42.7)	157 (45.2)	158 (45.5)	157 (45.2)	
Severe depression with recurrent episodes	87 (6.3)	31 (8.9)	18 (5.2)	19 (5.5)	19 (5.5)	
Laboratory examination						
Glucose, mg/dL, mean(IQR)	107.1 (94.0, 121.1)	93.3 (86.6, 104.2)	105.6 (93.1, 116.4)	110.5 (98.0, 123.0)	119.8 (108.5, 139.5)	< 0.001
Triglyceride,mean (IQR)	114.0 (77.0, 167.0)	62.0 (50.0, 74.0)	95.0 (83.1, 107.2)	136.0 (120.0, 155.5)	230.5 (183.5, 305.0)	< 0.001
TC, mean(IQR)	158.0 (134.0, 190.0)	151.0 (126.5, 180.0)	158.0 (133.0, 189.0)	160.0 (138.5, 189.5)	165.0 (137.0, 200.5)	< 0.001
HDL, mean(IQR)	46.0 (34.0, 59.0)	53.0 (40.0, 65.0)	50.0 (37.0, 62.2)	44.0 (33.0, 54.5)	39.0 (29.0, 51.0)	< 0.001
LDH, mean(IQR)	88.0 (68.0, 114.0)	80.0 (62.5, 103.5)	90.0 (67.5, 113.0)	92.0 (73.0, 113.0)	92.0 (70.0, 121.0)	< 0.001
ALT, mean(IQR)	26.0 (15.5, 53.1)	23.0 (15.0, 49.0)	21.0 (14.3, 46.6)	27.0 (15.6, 58.4)	31.5 (19.0, 61.4)	< 0.001
AST, mean(IQR)	30.2 (19.0, 60.0)	31.0 (19.0, 56.5)	27.0 (17.7, 47.5)	30.0 (19.6, 65.6)	34.0 (21.0, 69.9)	< 0.001
Serum creatinine, mg/dL,mean(IQR)	0.8 (0.6, 1.0)	0.8 (0.6, 0.9)	0.8 (0.6, 1.0)	0.8 (0.6, 1.0)	0.8 (0.6, 1.2)	0.053
Concomitant medication						
Antidepressant, n (%)	1046 (75.4)	249 (71.8)	269 (77.5)	265 (76.4)	263 (75.8)	0.318
Comorbidities						
Myocardial infarct, n (%)	56 (4.0)	8 (2.3)	10 (2.9)	13 (3.7)	25 (7.2)	0.005
Congestive heart failure, n (%)	136 (9.8)	32 (9.2)	35 (10.1)	34 (9.8)	35 (10.1)	0.978
Cerebrovascular disease, n (%)	183 (13.2)	50 (14.4)	43 (12.4)	45 (13)	45 (13)	0.879
Atrial fibrillation, n (%)	164 (11.8)	43 (12.4)	38 (11)	33 (9.5)	50 (14.4)	0.224
Hypertension, n (%)	575 (41.4)	114 (32.9)	154 (44.4)	143 (41.2)	164 (47.3)	< 0.001
Ischemic Stroke, n (%)	86 (6.2)	20 (5.8)	23 (6.6)	23 (6.6)	20 (5.8)	0.931
Rheumatic disease, n (%)	44 (3.2)	6 (1.7)	13 (3.7)	12 (3.5)	13 (3.7)	0.363
Peptic ulcer disease, n (%)	40 (2.9)	8 (2.3)	6 (1.7)	11 (3.2)	15 (4.3)	0.192
Liver disease, n (%)	256 (18.4)	71 (20.5)	52 (15)	60 (17.3)	73 (21)	0.135
Renal disease, n (%)	115 (8.3)	29 (8.4)	27 (7.8)	30 (8.6)	29 (8.4)	0.981
Events						
LOS hospital, days (IQR)	8.4 (4.2, 16.9)	6.6 (3.6, 12.7)	7.5 (3.6, 15.7)	9.7 (5.7, 16.7)	12.8 (6.1, 23.6)	< 0.001
In-hospital mortality, n (%)	86 (6.2)	8 (2.3)	10 (2.9)	25 (7.2)	43 (12.4)	< 0.001
365-day mortality, (%)	271 (19.5)	51 (14.7)	58 (16.7)	70 (20.2)	92 (26.5)	< 0.001
5-year mortality, (%)	358 (25.8)	67 (19.3)	88 (25.4)	90 (25.9)	113 (32.6)	0.001

TyG index: Q1 (6.88-8.29), Q2 (8.29-8.73), Q3 (8.73-9.17), Q4 (9.17-12.14).

Patients were further divided into two groups based on the number of TyG index measurements during hospitalization. **Table 2** showed the baseline features of the two groups before and after matching. After PSM, 532 patients were included in the study, of which 266 patients in the TyGVR group (receiving TyG measurements ≥2 times during hospitalization) were matched with 266 patients in the TyG group (receiving TyG measurements only once during hospitalization). The SMD of all variables was <0.10, indicating that the baseline variable distribution between the two groups is similar (**Table 2**), and the baseline variables were comparable between the two groups. The covariate balance after propensity score matching is visualized through Love plot (see additional file: **Supplementary Figure S1**).

**Table 2:** Baseline characteristics between two groups before and after PSM.

Variables	Entire cohort(n=1388)		SMD	PSM cohort(n=532)		SMD
	TyG was measured 1 time during hospital stay (n=1120)	TyG was measured≥2 times during hospital stay (n=268)		TyG was measured 1 time during hospital stay (n=266)	TyG was measured≥2 times during hospital stay(n=266)	
Demographics						
Age, year	54.80 (18.40)	52.32 (16.78)	0.141	53.20 (17.68)	52.30 (16.82)	0.052
Gender, Male, n (%)	412 (36.8)	111 (41.4)	0.095	110 (41.4)	110 (41.4)	<0.001
Race, white, n (%)	822 (73.4)	181 (67.5)	0.129	189 (71.1)	180 (67.7)	0.073
BMI, kg/m2	28.26 (8.18)	27.74 (8.02)	0.065	27.34 (7.86)	27.77 (8.04)	0.054
Depression level			0.244			0.048
Depression	550 (49.1)	131 (48.9)		131 (49.2)	131 (49.2)	
Severe depression with single episode	489 (43.7)	131 (48.9)		127 (47.7)	129 (48.5)	
Severe depression with recurrent episodes	81 (7.2)	6 (2.2)		8 (3.0)	6 (2.3)	
Laboratory examination						
TC, mean(IQR)	165.07 (53.52)	158.17 (55.56)	0.126	154.98 (54.37)	158.34 (55.71)	0.061
HDL, mean(IQR)	48.49 (19.54)	43.78 (18.30)	0.249	44.14 (19.73)	43.86 (18.34)	0.015
LDH, mean(IQR)	91.80 (35.57)	89.02 (38.86)	0.075	85.84 (34.51)	89.17 (38.91)	0.09
ALT, mean(IQR)	114.22 (503.10)	133.75 (511.32)	0.039	137.44 (579.61)	132.11 (512.30)	0.01
AST, mean(IQR)	130.10 (576.51)	167.33 (644.27)	0.061	174.58 (712.58)	160.68 (637.24)	0.021
Serum creatinine, mg/dL, mean(IQR)	1.00 (0.82)	0.97 (0.96)	0.026	1.01 (0.86)	0.97 (0.96)	0.055
Concomitant medication						
Antidepressant, n (%)	849 (75.8)	197 (73.5)	0.053	199 (74.8)	196 (73.7)	0.026
Comorbidities						
Myocardial infarct, n (%)	43 (3.8)	13 (4.9)	0.05	12 (4.5)	12 (4.5)	<0.001
Congestive heart failure, n (%)	117 (10.4)	19 (7.1)	0.119	16 (6.0)	19 (7.1)	0.046
Cerebrovascular disease, n (%)	171 (15.3)	12 (4.5)	0.368	8 (3.0)	12 (4.5)	0.079
Atrial fibrillation, n (%)	131 (11.7)	33 (12.3)	0.019	34 (12.8)	31 (11.7)	0.034
Hypertension, n (%)	469 (41.9)	106 (39.6)	0.047	91 (34.2)	104 (39.1)	0.102
Ischemic Stroke, n (%)	78 (7.0)	8 (3.0)	0.184	5 (1.9)	8 (3.0)	0.073
Rheumatic disease, n (%)	35 (3.1)	9 (3.4)	0.013	10 (3.8)	8 (3.0)	0.042
Peptic ulcer disease, n (%)	31 (2.8)	9 (3.4)	0.034	10 (3.8)	9 (3.4)	0.02
Liver disease, n (%)	196 (17.5)	60 (22.4)	0.123	61 (22.9)	58 (21.8)	0.027
Renal disease, n (%)	102 (9.1)	13 (4.9)	0.168	11 (4.1)	13 (4.9)	0.036
Events						
LOS hospital, days (IQR)	7.0 (3.8, 14.0)	17.8 (10.5, 30.7)	< 0.001	7.2 (4.1, 14.8)	17.8 (10.5, 30.8)	< 0.001
In-hospital mortality, n (%)	62 (5.5)	24 (9)	0.037	14 (5.3)	23 (8.6)	0.125
365-day mortality, (%)	197 (17.6)	74 (27.6)	< 0.001	46 (17.3)	73 (27.4)	0.005
5-year mortality, (%)	267 (23.8)	91 (34)	< 0.001	64 (24.1)	90 (33.8)	0.013

PSM, propensity score matching.

### Association of TyG index and mortality

The entire cohort exhibited a 5-year all-cause mortality rate of 25.8% (358/1388), with rates of 23.8% (267/1120) and 34% (91/268) in the TyG and TyGVR groups, respectively (**Table 2**). In the multivariate Cox regression analysis, we adjusted three models, which included covariates that showed significant differences (P<0.05) in the univariate analysis (see additional file: **Supplementary Table S2**).

In the entire cohort, TyG increased the 5-year risk of all-cause mortality in patients with depression in a multivariate Cox regression model (HR, 1.31; 95% CI, 1.1-1.56; P=0.002) (**Table 3**). When TyG was used as a categorical variable, Quartile 4 was associated with a 61% increased risk of 5-year all-cause mortality in depressed patients compared to Quartile 1 (HR, 1.61; 95% CI, 1.15-2.25; P=0.005) (**Table 3**). The trend was statistically different (HR, 1.16; 95% CI, 1.04~1.29; P=0.007). In terms of secondary endpoints, in multivariate Cox regression analysis, TyG index was associated with increased in-hospital mortality and 1-year all-cause mortality, with statistical differences, and HRs were 1.7 (95%CI 1.18~2.45; P=0.004) and 1.39 (95% CI 1.14-1.7; P=0.001), respectively (**Table 3**).

**Table 3:** Multivariable Cox regression analysis for association of TyG index and mortality.

Categories	Model I		Model II		Model III	
	HR (95%CI)	P-value	HR (95%CI)	P-value	HR (95%CI)	P-value
Primary outcome						
5-year mortality						
TyG index (continuous)	1.36 (1.18~1.56)	<0.001	1.4 (1.21~1.62)	<0.001	1.31 (1.1~1.56)	0.002
TyG index (quartiles)						
Quartile 2:1	1.35 (0.98~1.85)	0.065	1.25 (0.91~1.72)	0.173	1.24 (0.89~1.71)	0.203
Quartile 3:1	1.42 (1.04~1.95)	0.029	1.39 (1.01~1.91)	0.042	1.25 (0.9~1.74)	0.189
Quartile 4:1	1.89 (1.4~2.56)	<0.001	1.94 (1.43~2.63)	<0.001	1.61 (1.15~2.25)	0.005
P for trend	1.22 (1.11~1.34)	<0.001	1.24 (1.12~1.36)	<0.001	1.16 (1.04~1.29)	0.007
Secondary outcomes						
In-hospital mortality						
TyG index (continuous)	1.78 (1.31~2.42)	<0.001	1.67 (1.22~2.28)	0.001	1.7 (1.18~2.45)	0.004
TyG index (quartiles)						
Quartile 2:1	0.97 (0.38~2.47)	0.955	0.96 (0.38~2.45)	0.933	1.16 (0.45~3.02)	0.761
Quartile 3:1	2.08 (0.94~4.63)	0.072	2.04 (0.92~4.55)	0.081	2.31 (1~5.35)	0.051
Quartile 4:1	2.95 (1.38~6.29)	0.005	2.67 (1.25~5.73)	0.011	2.79 (1.23~6.3)	0.014
P for trend	1.53 (1.23~1.91)	<0.001	1.47 (1.18~1.84)	0.001	1.44 (1.14~1.83)	0.002
1-year mortality						
TyG index (continuous)	1.4 (1.2~1.65)	<0.001	1.43 (1.21~1.69)	<0.001	1.39 (1.14~1.7)	0.001
TyG index (quartiles)						
Quartile 2:1	1.16 (0.79~1.68)	0.452	1.07 (0.74~1.56)	0.718	1.1 (0.75~1.62)	0.634
Quartile 3:1	1.44 (1.01~2.07)	0.046	1.4 (0.98~2.02)	0.066	1.36 (0.93~2.01)	0.115
Quartile 4:1	1.98 (1.41~2.79)	<0.001	2 (1.41~2.82)	<0.001	1.76 (1.19~2.6)	0.005
P for trend	1.26 (1.13~1.41)	<0.001	1.28 (1.14~1.43)	<0.001	1.22 (1.07~1.38)	0.002

Model I: did not adjust any variables.

Model II: adjusted for age, gender, race, BMI.

Model III:depression level, TC, HDL, LDH, serum creatinine, myocardial infarct, congestive heart failure, cerebrovascular disease, atrial fibrillation, hypertension, ischemic stroke, diabetes, liver disease, renal disease.

TyG index: Q1(6.88~8.29), Q2 (8.29~8.72), Q3 (8.72~9.17), Q4 (9.17~12.14).

**Figure 2:**
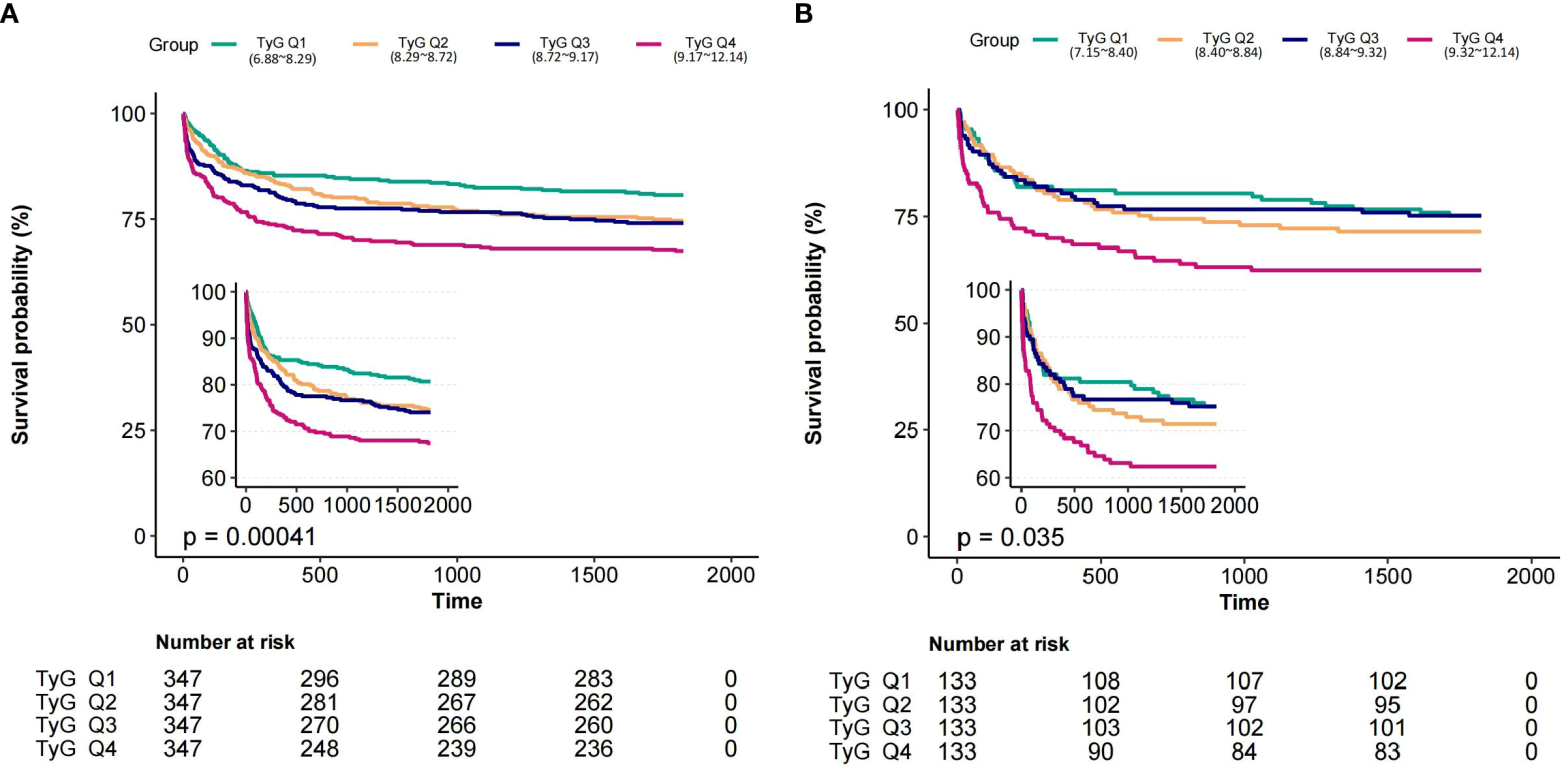
Kaplan-Meier curve showed the cumulative 5-year death probability according to the quartile of TyGVR index in entire **(A)** and PSM cohorts **(B)**. In entire cohort, TyGVR: Q1 (7.15~8.28), Q2 (8.28~8.63), Q3 (8.63~9.04), Q4 (9.04~11.11). In matched cohort TyGVR: Q1 (-0.3127~-0.0191), Q2 (-0.0191~0.0238), Q3 (0.0238~0.0707), Q4 (0.0707~0.3071) TyGVR, triglyceride-glucose index variability ratio.

**Figure 2** showed the Kaplan-Meier curve for evaluating 5-year all-cause mortality of depressed patients based on the TyG index. Patients with higher TyG had significantly higher 5-year all-cause mortality in both the entire and matched cohorts (P=0.00041, P=0.035, respectively).

**Figure 3:**
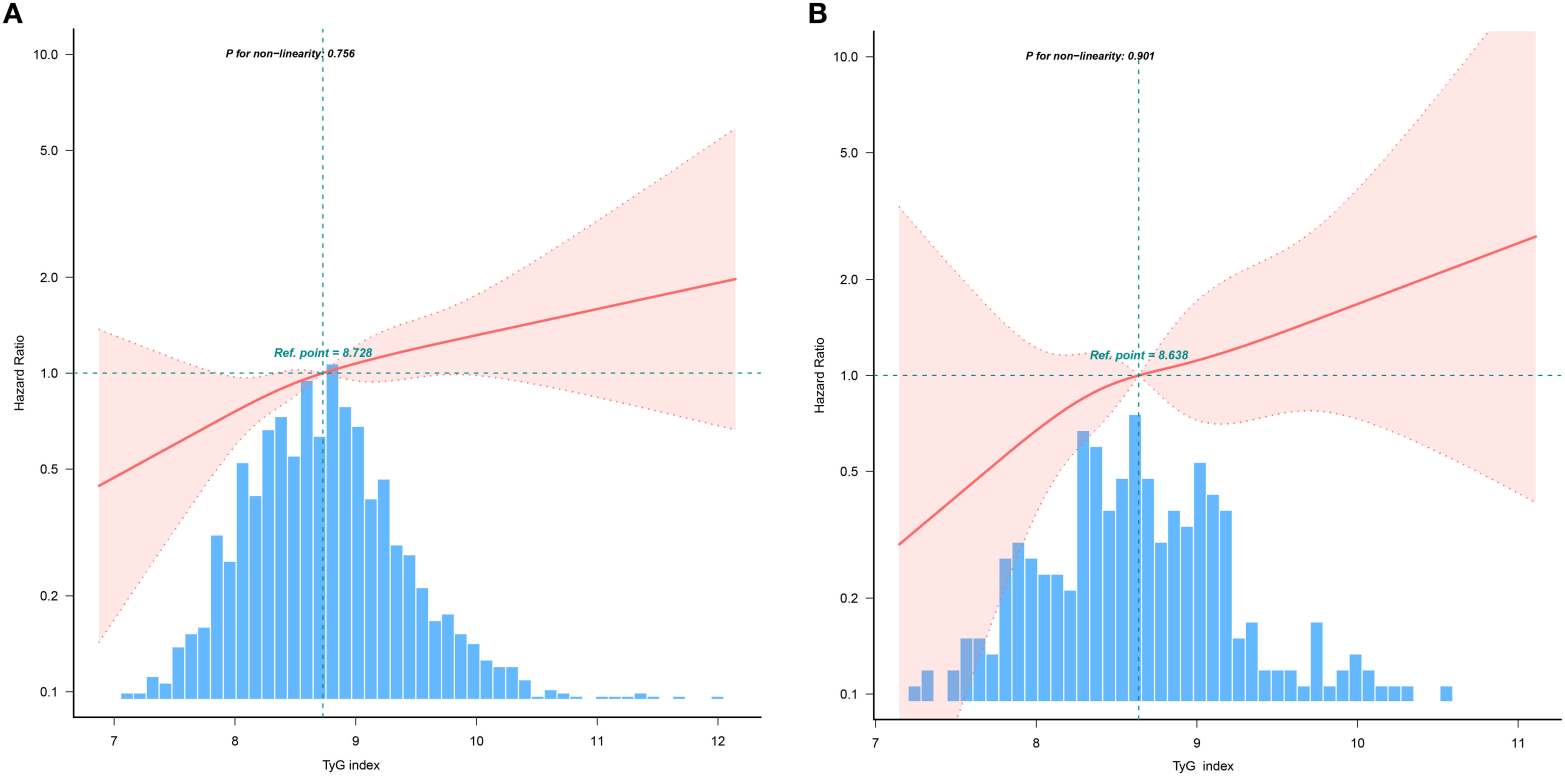
Potential non-linear relationship between TyG index and 5-year all-cause mortality. **(A)** TyG and 5-year all-cause mortality in entire cohort; **(B)** TyG and 5-year all-cause mortality in matched cohort.

Restricted cubic spline analysis showed a linear association between TyG and 5-year all-cause mortality, both in entire and matched cohort (P values for non-linear were>0.05, **Figure 3**).

**Figure 4:**
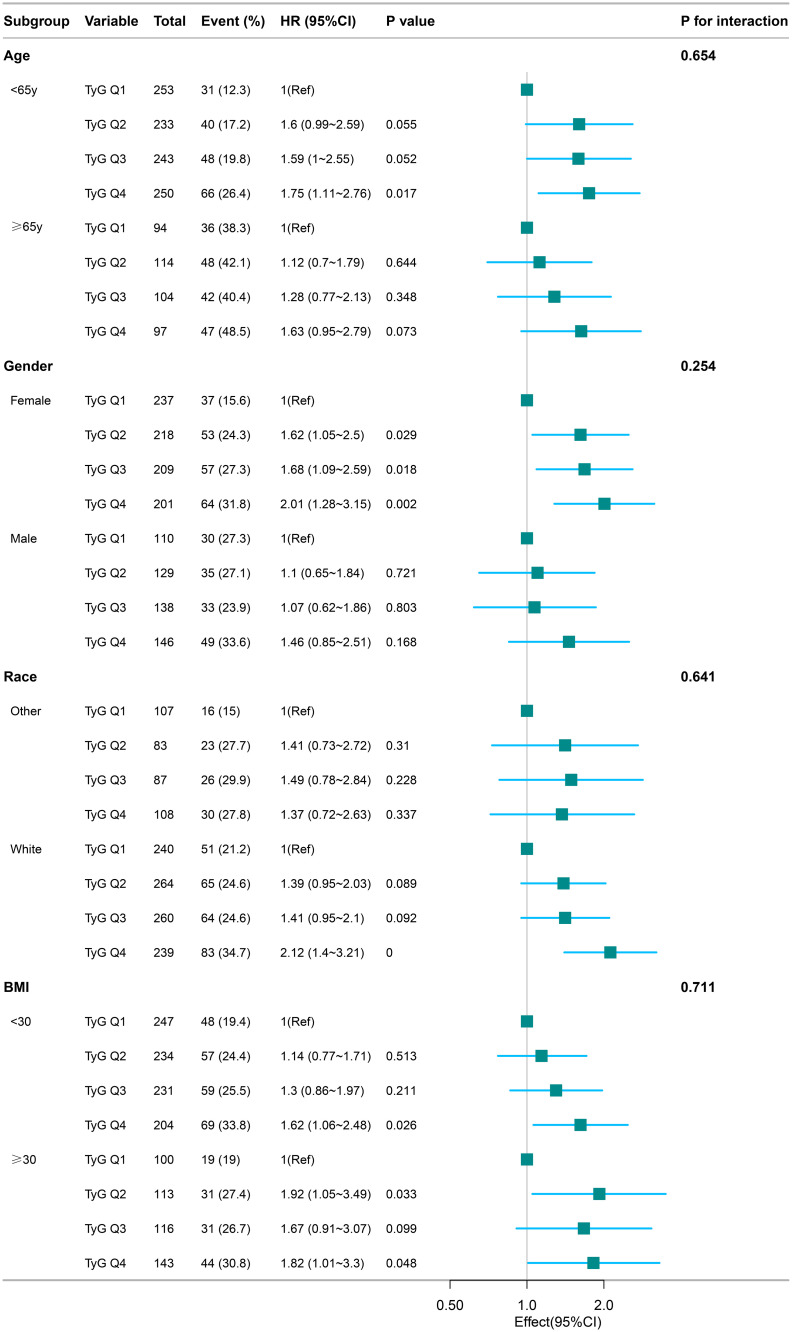
Subgroup analyses for the association of TyG index and 5-year death. Adjusted for depression level, TC, HDL, LDH, serum creatinine, congestive heart failure, cerebrovascular disease, ischemic stroke, diabetes, liver disease, renal disease.

A subgroup analysis was performed,we used age (< 65, ≥ 65 years), gender (female, male), race (white, other), and BMI (< 30, ≥ 30) as stratification variables to observe the effect values and generate a forest plot of data (**Figure 4**).We did not observe any significant interactions in all subgroups (P values of interactions were>0.05). Subgroup analysis showed that the relationship remained robust and reliable.

### Association of TyGVR and 5-year all−cause mortality

Restricted cubic spline analysis demonstrated a linear association between TyGVR and the risk of 5-year all-cause mortality (P for non-linear=0.953) (See additional file: **Figure 2S**); the higher the TyGVR value, the higher the risk of death. The association between the TyGVR index and 5-year all-cause mortality was consistent across all sub-populations (P values for interactions were>0.05) (See additional file: **Figure 3S**). The link remained robust and reliable, according to subgroup analysis. All the above analyses were carried out using matched data.

## Discussion

This study is the first to explore the association between TyG level and long-term risk of death in patients with depression. More critically, our study further focused on the impact of TyG changes on long-term mortality. The study findings indicated that even after adjusting for potential confounding factors, a high level of TyG was associated with an increase in 5-year all-cause mortality. Various stratification and sensitivity analyses revealed the connection to be constant. Furthermore, the risk of 5-year all-cause mortality escalates linearly with increasing levels of TyG and TyGVR. These findings have significant clinical implications. TyG is a simple and easily accessible biomarker that combines TyG index measurement with routine depression screening, and dynamic monitoring of TyG changes may help identify high-risk individuals early and reduce future mortality events.

A bidirectional causal relationship exists between insulin resistance (IR) and depression (34, 35). Individuals with IR risk factors (including type 2 diabetes, hypertension, obesity, etc.) have an increased risk of depression (21, 36–38). In the brain, IR can lead to reduced metabolism, altered blood vessels, dysregulated neuroplasticity, and structural changes, all of which can initiate or exacerbate depression (37–39). Results from an epidemiological study revealed that young adults and non-diabetic individuals with increased IR may be associated with depressive symptoms (40). Conversely, depression has also been shown to significantly increase the risk of developing T2DM and other metabolic complications such as peripheral insulin resistance, metabolic syndrome, and vascular disease (22, 41). A large meta-analysis revealed that IR was increased in depression during acute episodes (42). Insulin resistance is considered to be a status marker of depression and is associated with current but unrelieved major depressive disorder (43).

IR and disturbances in glucose and lipid metabolism have been linked to suicide attempts and ideation in depression (44). Prior research has demonstrated an association between higher blood sugar levels and dysthymia (45). Triglycerides have also been reported as a potential risk factor for depression (46). Some studies suggest that low cholesterol levels are related to impulsivity and an increased risk of suicide in individuals with depression (47–49), while another study indicates a connection between high cholesterol concentrations and suicidal behavior (50). The TyG index, calculated using triglyceride and fasting blood glucose levels, has emerged as a novel metric for evaluating insulin resistance in recent years (51). Shi et al. (25) analyzed 13,350 participants, including 1,001 individuals with depression, from the National Health and Nutrition Examination Survey (NHANES 2005-2018), reporting that each unit increase in the TyG index corresponds to a 28% increase in the likelihood of developing depression. In another study, a positive association was found between the TyG index and the progression of depression, particularly in men, older adults, and overweight individuals (52). Lee et al. (53) found that the TyG level of participants with suicidal ideation was significantly higher than that of individuals without suicidal ideation. However, in a study focusing on the Chinese population (26), the relationship between the TyG index and the risk of suicide attempt was non-linear, with a threshold effect observed in patients with major depressive disorder initially treated with the first drug. When the TyG index was greater than 9.29, a significant positive correlation was observed. These studies collectively suggest an association between the TyG index and depression severity. The recently demonstrated unique link between lipid dysregulation, depression, and cognition by Mehdi et al. (54) provides a compelling pathophysiological substrate for our findings, suggesting that the TyG index may capture a shared biological mechanism driving adverse outcomes in depression.

Apart from mortality resulting from suicide, the elevated TyG index and increased long-term mortality risk in patients diagnosed with depression may also be attributed to cerebrovascular and other vascular complications driven by insulin resistance (IR) and its downstream effects. IR contributes to the pathogenesis of ischemic stroke through multiple mechanisms. It interferes with insulin signaling, enhances chronic systemic inflammation, reduces insulin sensitivity, and accelerates atherosclerosis and late plaque formation by promoting foam cell formation (55). Furthermore, IR affects the metabolism of insulin-like growth factor-1 (IGF-1), insulin-like growth factor-2 (IGF-2) (56), cyclic guanosine monophosphate (cGMP), and nitric oxide (NO) (57), playing a key role in platelet adhesion, activation, and aggregation (58). These pathways ultimately lead to vascular occlusion and the pathogenesis of ischemic stroke. Consistent with this, Cui et al. demonstrated that the TyG index exerted a significant mediating effect on ischemic stroke events partly through inflammatory biomarkers (59). Underlying mechanisms may involve the polarization of macrophages and lymphocytes toward a pro-inflammatory phenotype, which can drive the progression of IR—a process potentially mediated by the renin-angiotensin-aldosterone system, sympathetic activation, and regulators of incretins (e.g., DPP-4) and immune responses (60, 61).

Numerous studies have confirmed that a high TyG index is associated with the prevalence and adverse prognosis of diseases such as diabetes, hypertension, coronary heart disease, and ischemic stroke (62–67). Moreover, as mentioned above, depression is also prone to multiple comorbidities, especially when it is poorly controlled. In our study, all these comorbidities were considered as covariates, and in order to minimize the interference of drugs on blood glucose or triglyceride, patients with diabetes mellitus, lipid-regulating drugs, and hypoglycemic drugs for other conditions were initially excluded. Consequently, compared to other studies, this study had a lower proportion of patients with cardiovascular complications, excluding hypertension. At baseline, the proportion of hypertension in the TyG index group ranged from Q1 to Q4, exceeding 40%, with the highest proportion in the Q4 group. However, the prevalence of other diseases in the TyG index was low, with no significant difference observed between the groups. Regrettably, due to data unavailability, the post-follow-up diagnosis of diabetes, metabolic syndrome, and cardiovascular and cerebrovascular diseases among the deceased patients remains unknown. Nonetheless, this does not affect the reliability of the conclusions of this study. By adjusting different variables, we established three models, all of which were consistent in direction and displayed no interaction in subgroup analysis.

Our results further verified the impact of TyG on the long-term prognosis of patients with depression. A key distinction lies in the primary outcome endpoint; previous studies focused on suicidal ideation or attempts (26, 53), whereas our study considered all-cause mortality, thus reducing the influence of subjective factors. In addition, the TyG index trajectory of depressed patients was further analyzed in this studyand to our knowledge, there has been no relevant exploration for depressed people. Insulin sensitivity changes over time, and IR was closely related to inflammation and stress immune responses to severe illness and its severity (68). Previous studies have explored the ability of longitudinal changes in TyG index over time to affect diseases (23, 66, 67, 69, 70), but there is currently no relevant research on its impact on depression. Our study revealed that high TyGVR values were associated with an increased risk of long-term mortality in patients with depression. This finding suggests that dynamic, real-time monitoring of the TyG index and risk assessment could be integrated into clinical practice, particularly for patients with severe conditions. However, due to the relatively short monitoring period (data collection only during hospitalization) and the small sample size (only 266 cases underwent two or more tests during hospitalization), the association between TyGVR and poor long-term prognosis in depressed patients warrants further exploration in future studies.

## Study limitations

This study has several limitations. First, its retrospective, single-database design limits generalizability and, despite statistical adjustments, cannot preclude residual confounding by unmeasured variables (e.g., lifestyle, socioeconomic factors) or establish causality. Second, the statistical power for key analyses was limited. Most notably, the subgroup with repeated TyG measurements was small (n=266), potentially inflating Type II error risk for variability analyses. Similarly, the sample lacked power to stratify by depression severity or relapse frequency. Third, the absence of longitudinal metabolic data during follow-up precludes investigation into mediating pathways. Fourth, despite statistical adjustment for TyG-related comorbidities, our study lacked a non-depressed control group. Therefore, we cannot rule out that the observed association exists in a broader population and is not unique to depression. Future comparative studies with matched cohorts are needed to conclusively determine if depression confers a unique susceptibility.

Despite these limitations, our findings underscore the prognostic value of the TyG index and highlight critical directions for future research. Prospective studies featuring dynamic monitoring of metabolic (e.g., TyG index) and neuroendocrine (e.g., HPA axis biomarkers like cortisol) parameters are warranted to dissect their temporal and mechanistic relationships with mortality in depression.

## Conclusions

Our findings suggested that there was an independent association between the TyG index and long-term mortality risk in patients with depression. The TyG index may be a useful biomarker for risk stratification and prognosis in patients with depression. In addition, the changes in TyG index may provide more valuable information for identifying high-risk all-cause mortality patients, but larger sample size studies are still needed to confirm this. This study further clarified the role of IR in patients with depression and provides a basis for future multi-center studies on depression and TyG index.

## Data Availability

The original contributions presented in the study are included in the article/**Supplementary Material**. Further inquiries can be directed to the corresponding author.
