# Supplementary material for: The association of the triglyceride-glucose index and its changes with 5-year all-cause mortality in patients with depression
Source: Front Psychiatry. 2025 Oct 8;16:1672186. doi: 10.3389/fpsyt.2025.1672186 (PMC12540422; doi:10.3389/fpsyt.2025.1672186)

**Supplementary material**

**Table S1.** Missing statistics

| **Variable** | **Miss.freq** | | **Miss.percentage%** |
| --- | --- | --- | --- |
| Creatinine, avg | 9 | 0.6484 | |
| AST, avg | 264 | 19.0202 | |
| ALT, avg | 267 | 19.2363 | |
| BMI | 349 | 25.1441 | |
| TC, avg | 614 | 44.2363 | |
| HDL, avg | 647 | 46.6138 | |
| LDL, avg | 660 | 47.5504 | |
| TSH, avg | 779 | 56.1239 | |
| Free thyroxine, avg | 1279 | 92.147 | |
| Thyroxine, avg | 1321 | 95.1729 | |
| Triiodothyronine, avg | 1349 | 97.1902 | |
| LDH, avg | 1352 | 97.4063 | |
| Thyroglobulin, avg | 1381 | 99.4957 | |
| Thyroid peroxidase antibodies, avg | 1381 | 99.4957 | |
| Anti-thyroglobulin antibodies, avg | 1388 | 100 | |

TC, total cholesterol. HDL, Low-density lipoprotein. LDL, Low-density lipoprotein. TSH, thyroid stimulating hormone. LDH, lactate dehydrogenase.

**Table S2.** Analysis of the impact of potential confounders of 5-year-mortality association after propensity score matching

|  | **HR(95%CI)** | **P(Wald's test)** |
| --- | --- | --- |
| Age, year | 1.03 (1.02,1.04) | < 0.001 |
| Gender, Male, n (%) | 1.28 (0.93,1.75) | 0.13 |
| Race, n (%) | 1.04 (0.74,1.47) | 0.817 |
| BMI, kg/m2 | 1.0061 (0.9872,1.0255) | 0.529 |
| Marital status |  | 0.037 |
| Single vs other | 0.55 (0.3,0.99) | 0.045 |
| Married vs other | 0.72 (0.4,1.28) | 0.262 |
| Divorced vs other | 0.62 (0.3,1.29) | 0.205 |
| Widowed vs other | 1.33 (0.64,2.76) | 0.441 |
| **Depression level** |  | 0.46 |
| Severe depression with single episode vs Depression | 1.18 (0.85,1.62) | 0.321 |
| Severe depression with recurrent episodes vs Depression | 0.72 (0.23,2.28) | 0.576 |
| Glucose, mg/dL (IQR) | 1.0053 (1.0028,1.0078) | < 0.001 |
| Triglyceride (IQR) | 1.0001 (0.9994,1.0008) | 0.837 |
| TC (IQR) | 0.9952 (0.9918,0.9986) | 0.006 |
| HDL (IQR) | 0.97 (0.96,0.98) | < 0.001 |
| LDH (IQR) | 0.9964 (0.9919,1.0009) | 0.113 |
| ALT (IQR) | 0.9999 (0.9995,1.0002) | 0.446 |
| AST (IQR) | 1 (0.9998,1.0002) | 0.853 |
| Serum creatinine, mg/dL (IQR) | 1.28 (1.13,1.46) | < 0.001 |
| Antidepressant, n (%) | 0.87 (0.61,1.24) | 0.434 |
| Myocardial infarct, n (%) | 1.86 (1.01,3.43) | 0.048 |
| Congestive heart failure, n (%) | 1.72 (1.01,2.93) | 0.046 |
| Peripheral vascular disease, n (%) | 1.66 (0.9,3.06) | 0.107 |
| Cerebrovascular disease, n (%) | 1.13 (0.5,2.56) | 0.769 |
| Atrial fibrillation, n (%) | 2.53 (1.73,3.7) | < 0.001 |
| Hypertension, n (%) | 1.99 (1.45,2.74) | < 0.001 |
| Ischemic Stroke, n (%) | 0.85 (0.27,2.66) | 0.779 |
| Chronic pulmonary disease, n (%) | 1.46 (1.04,2.05) | 0.031 |
| Rheumatic disease, n (%) | 0.95 (0.39,2.31) | 0.903 |
| Peptic ulcer disease, n (%) | 1.4 (0.66,2.99) | 0.382 |
| Liver disease, n (%) | 1.68 (1.19,2.38) | 0.003 |
| Renal disease, n (%) | 2.03 (1.13,3.66) | 0.019 |
| TyG | 1.29 (1.06,1.58) | 0.012 |
| TyG group |  | 0.045 |
| TyG (Q2:Q1) | 1.17 (0.73,1.86) | 0.515 |
| TyG (Q3:Q1) | 1.02 (0.63,1.65) | 0.949 |
| TyG (Q4:Q1) | 1.73 (1.11,2.68) | 0.015 |

TC, total cholesterol. HDL, Low-density lipoprotein. LDL, Low-density lipoprotein. TSH, thyroid stimulating hormone. LDH, lactate dehydrogenase. TyG, index triglyceride glucose index.

**Figure S1.** Covariate Balance Assessment (Love Plot)


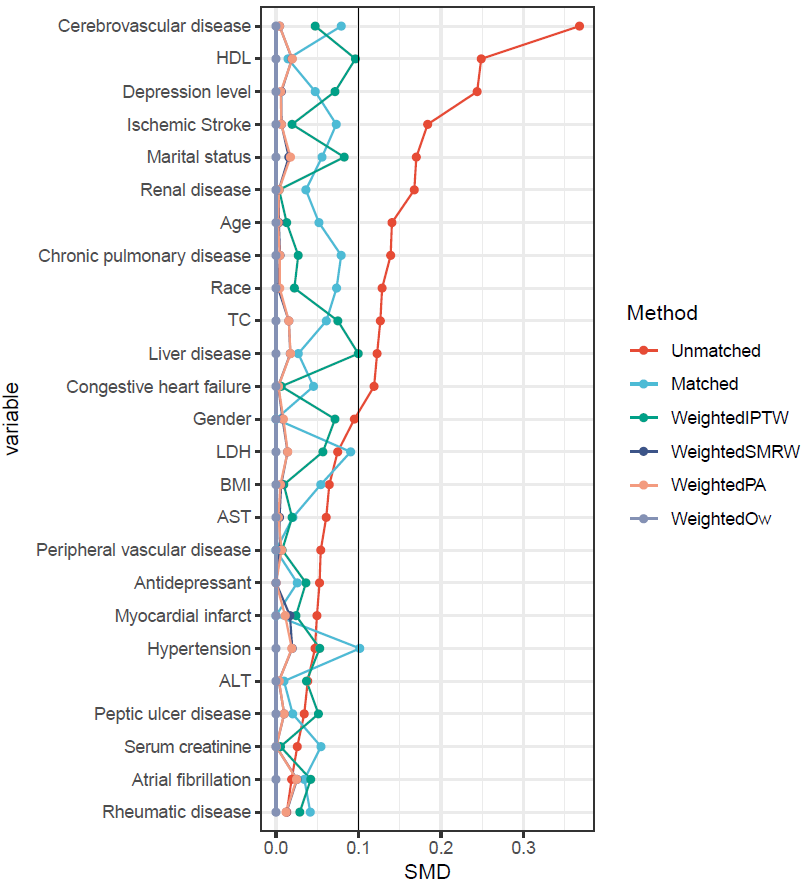


**Figure 2S.** Potential non-linear relationship between TyGVR index and 5-year death.


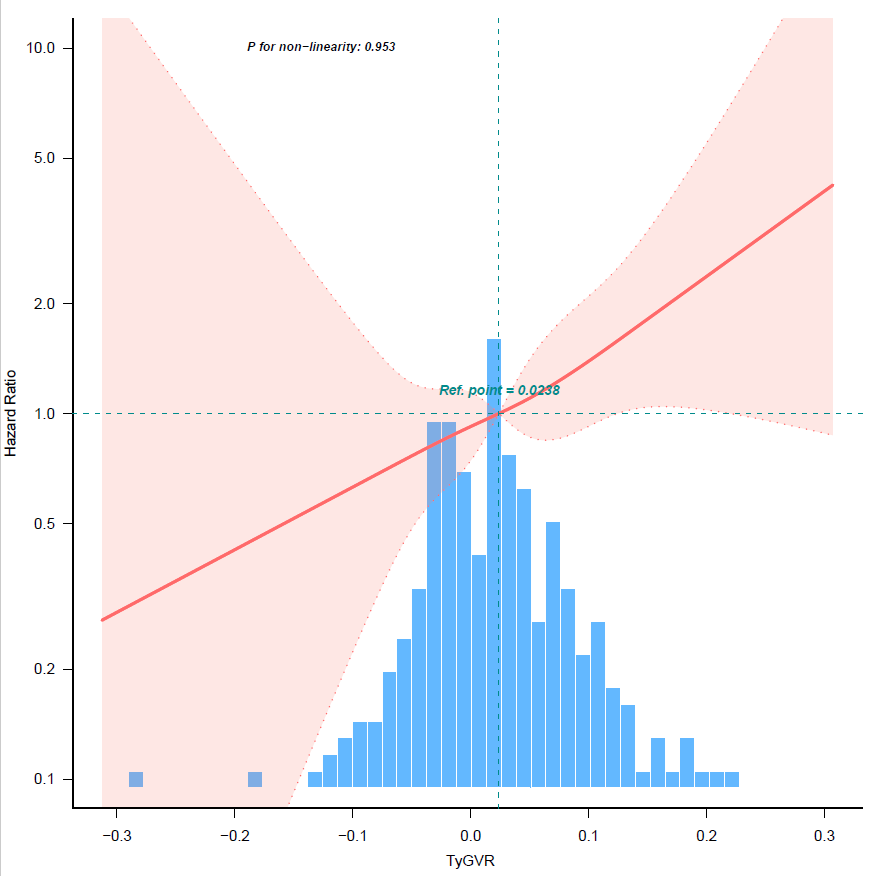


**Figure 3S.** Subgroup analyses for the association of TyGVR index and 5-year death.


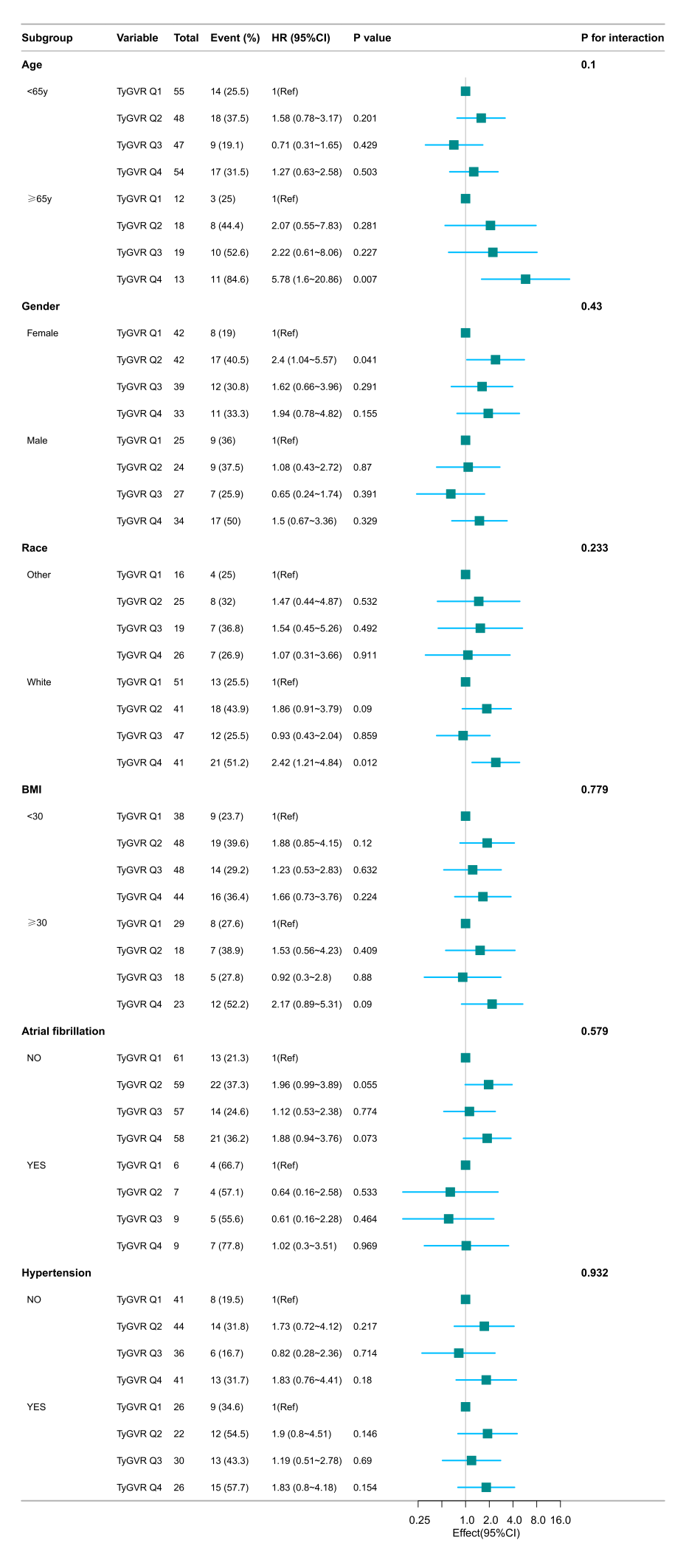

Supplement: Supplementary file 1 [file Supplementaryfile1.docx]
